# Altered methylation and expression of ER-associated degradation factors in long-term alcohol and constitutive ER stress-induced murine hepatic tumors

**DOI:** 10.3389/fgene.2013.00224

**Published:** 2013-10-31

**Authors:** Hui Han, Jay Hu, Mo Y. Lau, Min Feng, Lydia M. Petrovic, Cheng Ji

**Affiliations:** ^1^GI/Liver Division, Department of Medicine, Keck School of Medicine, University of Southern CaliforniaLos Angeles, CA, USA; ^2^Department of Pathology, Keck School of Medicine, University of Southern CaliforniaLos Angeles, CA, USA

**Keywords:** alcohol, aging, unfolded protein response, ERAD, hepatocellular tumorigenesis

## Abstract

Mortality from liver cancer in humans is increasingly attributable to heavy or long-term alcohol consumption. The mechanisms by which alcohol exerts its carcinogenic effect are not well understood. In this study, the role of alcohol-induced endoplasmic reticulum (ER) stress response in liver cancer development was investigated using an animal model with a liver knockout (KO) of the chaperone BiP and under constitutive hepatic ER stress. Long-term alcohol and high fat diet feeding resulted in higher levels of serum alanine aminotransferase, impaired ER stress response, and higher incidence of liver tumor in older (aged 16 months) KO females than in either middle-aged (6 months) KOs or older (aged 16 months) wild type females. In the older KO females, stronger effects of the alcohol on methylation of CpG islands at promoter regions of genes involved in the ER-associated degradation (ERAD) were also detected. Altered expression of ERAD factors including derlin 3, Creld2 (cysteine-rich with epidermal growth factor-like domains 2), Herpud1 (homocysteine-inducible, endoplasmic reticulum stress-inducible, ubiquitin-like domain member), Wfs1 (Wolfram syndrome gene), and Yod1 (deubiquitinating enzyme 1) was co-present with decreased proteasome activities, increased estrogen receptor α variant (ERα36), and enhanced phosphorylations of ERK1/2 (extracellular signal-regulated protein kinases 1 and 2) and STAT3 (the signal transducers and activators of transcription) in the older KO female fed alcohol. Our results suggest that long-term alcohol consumption and aging may promote liver tumorigenesis in females through interfering with DNA methylation and expression of genes involved in the ERAD.

## INTRODUCTION

Liver cells are rich in the essential organelle-endoplasmic reticulum (ER), which assumes synthesis of a large amount of secretory and membrane proteins and lipids, maintains intracellular calcium homeostasis, and detoxifies drugs (Dara et al., 2011; Cao and Kaufman, 2013). For the protein synthesis and modifications, the ER ensures correct protein folding and maturation. Unfolded proteins are normally retained in the ER and targeted for retro translocation to the cytoplasm for rapid removal through the ER-associated protein degradation (ERAD). Malfunction of the ER leads to ER stress and accumulation of unfolded proteins triggering the unfolded protein response (UPR). The UPR is essentially mediated by molecular chaperones such as the glucose-regulated protein 78 (GRP78/BiP), which interact with three ER membrane resident stress sensors: inositol-requiring enzyme-1 (IRE1α), activating transcription factor-6 (ATF6), and protein kinase R (PKR)-like eukaryotic initiation factor 2α kinase (PERK; Walter and Ron, 2011). The UPR reduces protein translation, enhances protein folding capacity, and accelerates degradation of unfolded proteins, restoring ER homeostasis. However, persistent or prolonged UPR leads to impaired hepatic lipid synthesis, aberrant immune response, and eventually an attempt to eliminate the over-stressed cells, causing liver injuries (Zhang, 2010; Fu et al., 2012).

Alcohol is the most socially accepted drug that is mainly metabolized in the liver. Alcohol is oxidized by alcohol dehydrogenase (ADH) or cytochrome P450IIE1 (CYP2E1) to acetaldehyde. Acetaldehyde dehydrogenase (ALDH) converts acetaldehyde to acetate which enters the circulation. Alcohol overdose leads to overproduction of highly reactive acetaldehyde, reactive oxygen species (ROS) and intracellular NADH, all of which collectively play etiological roles in alcoholic pathologies (Zakhari, 2006; Zakhari and Li, 2007; Gao and Bataller, 2011). Growing evidence indicates that alcohol-induced liver ER stress contributes to liver disease (Ji, 2012). ER proliferation and liver injury is associated with microsomal alcohol oxidations by CYP2E1 in rats and humans (Cinti et al., 1973; Lieber, 1987). Multiple alcohol consumption-related factors including free radicals, acetaldehyde, toxic lipid species, oxidative stress, excessive homocysteine or *S*-adenosyl methionine (SAH) from impaired one carbon metabolism, disruption of calcium homeostasis, and insulin resistance are reported to disturb ER homeostasis and induce hepatic ER stress in cultured hepatocytes as well as in the livers of several species including mouse, rat, minipigs, zebrafish, and humans (Ji and Kaplowitz, 2003; Nishitani and Matsumoto, 2006; Passeri et al., 2009; Esfandiari et al., 2010; Magne et al., 2011; Galligan et al., 2012; Kao et al., 2012; Longato et al., 2012; Ramirez et al., 2012, 2013). However, the importance of alcohol-induced ER stress in liver injury may depend on other genetic and environmental factors, patterns of alcohol exposure, and stages of liver disease (Ji, 2012). Alcohol-induced liver cirrhosis and hepatocellular carcinogenesis (HCC) is often enhanced by high fat diet (HFD) feeding or by aberrant epigenetic factors such as methylation of genome DNA (Seitz and Becker, 2007; Shukla et al., 2008; Philibert et al., 2012; Loomba et al., 2013; Tsuchishima et al., 2013). It is unclear whether the alcohol-induced ER stress is also involved in the development of liver cancer and whether epigenetic modifications of ER stress factors contribute to alcohol-induced advanced liver injury. Considering that epigenetic inactivation of genes play a critical role in many important human diseases such as cancer and that methylation of CpG islands of the genomic DNA is in general a core mechanism for epigenetic inactivation of genes (Rakyan et al., 2011), we hypothesize that alcohol consumption affects DNA methylation of genes pertinent to the UPR/ER stress response and we tested the hypothesis in a liver tumor-prone mouse model under constitutive hepatic ER stress.

## MATERIALS AND METHODS

### ANIMAL EXPERIMENTS

Mouse models with a liver-specific deletion of the immunoglobulin heavy chain-binding protein (BiP), also known as glucose-regulated protein 78 (Grp78) were previously created through the LoxP-Cre strategy (Luo et al., 2006; Ji et al., 2011). Briefly, the established BiP floxed females (*BiP*^f/f^) were crossed with male mice carrying the *Cre* transgene under the control of the rat albumin promoter (*Alb-Cre*). The resulting heterozygous mice carrying the floxed alleles and the *Alb-Cre* gene were back-crossed with the BiP floxed founders to yield mice with liver-specific BiP deletion. The littermates carrying homozygous floxed alleles without the *Alb-Cre* gene were used as wild type (WT) controls. PCR genotyping with tail or liver genomic DNA was performed to distinguish BiP alleles of WT and knockout (KO). The presence of the *Alb-Cre* transgene was determined by duplex quantitative PCR using *Cre*-specific primers. Animal breeding, genotyping, daily inspection, and maintenance of the colonies were described previously (Ji et al., 2011; Lau et al., 2013). The animals were grouped into two age groups. One group was 6–8 months old termed middle-aged (Mid) group; the other group was 12–16 months old termed older (Old) group that corresponds to humans aged of approximately 50. The older KOs with suspected spontaneous liver tumor development without alcohol were excluded from the experiments. Animals that were moribund, unable to move or failure to respond to gentle stimuli, with labored breathing or diarrhea, and inability to eat and drink were eliminated from the experiments. For long-term alcohol treatment, mice were fed orally a liquid HFD (AIN-93G #710301; Dyets, Inc., Bethlehem, PA, USA) mixed with alcohol at a dose of 4 g alcohol/kg body weight or an isocaloric HFD (#710301) without alcohol for 12 months. Pair feeding was conducted by feeding the alcohol group in the first day of the experiment and by measuring amount of alcohol diet consumed by each animal in the next day, which was used to calculate isocalorically matched control diet for the control group. Occasionally, there were a couple of hours’ waiting time for the control mice since some of the mice tended to consume the control diet without alcohol more and faster than the diet with alcohol. All animals were treated in accordance with the Guide for Care and Use of Laboratory Animals approved by a local committee for animal care and use.

### PARAMETERS OF LIVER INJURY

At the time of killing, serum samples were collected and liver tissues were either snap frozen in liquid nitrogen and stored at -80°C or fixed immediately for histological staining. Serum alanine aminotransferase (ALT) and liver histology for hematoxylin and eosin (H&E) staining and immunohistochemistry were described previously (Kao et al., 2012). Histological changes were checked by a pathologist blinded to the genotypes. The Betazoid DAB Chromogen kit and ancillary reagents (BioCare Medical, CA, USA) were used for the immunohistochemistry. Primary antibodies against the molecular marker of proliferation Ki-67 were from Santa Cruz Biotechnology Inc. (Santa Cruz, CA, USA). Hepatocytes stained positive with anti-Ki-67 were counted under a microscope at 100× magnification.

### IMMUNOBLOTTING OF LIVER PROTEINS AND PROTEASOME ACTIVITIES

Proteins (whole or nuclear) were extracted respectively from WT liver tissues, KO without liver tumors and the normal liver portion and the tumor portion from tumor bearing livers of KOs, which were analyzed according to the methods described previously (Kao et al., 2012; Lau et al., 2013). Immunoblotting was conducted using horseradish peroxidase-labeled secondary antibodies (1:2000 dilutions), in which the intensity of protein bands on the immunoblots was quantified with the NIH software, ImageJ. Primary antibodies against BiP, CCAAT-enhancer-binding proteins (C/EBP) homologous protein (CHOP; sc-7351), ATF6 (sc-22799), GRP94, protein disulfide isomerase (PDI), cysteine-rich with epidermal growth factor (EGF)-like domains 2 (Creld2), Der1p-like protein (derlin), cyclin D, estrogen receptor α, homocysteine-induced ER protein (HERP), phosphorylated extracellular signal-regulated protein kinases 1 (p-ERK1/2), and phosphorylated signal transducers and activators of transcription 3 (p-STAT3) were from Santa Cruz Biotechnology Inc. Primary antibodies against the transcription activator 4 (ATF4) were from Aviva System (San Diego, CA, USA). Primary antibodies against glyceraldehyde 3-phosphate dehydrogenase (GAPDH) were from Millipore (Billerica, MA, USA). Primary antibodies against β-actin were from Sigma. Proteasome activities were assessed with the 20S Proteasome Activity Assay Kit from MILLIPORE (Billerica, MA, USA) and the relative fluorescent units were recorded with the Omega Microplate Readers from BGM LABTECH (Gary, NC, USA) using 355/460 nm filter set.

### PCR ANALYSIS OF PROMOTER METHYLATION

For analysis of promoter methylation of ER stress marker genes, genomic DNA was extracted from the mouse liver tissues using the QIAGEN DNeasy Tissue Kit (Valencia, CA, USA). Methylation was analyzed with a methylation promoter PCR kit (Panomics; Fremont, CA, USA). Briefly, the isolated genomic DNA was digested with *Mse*I, and the resulting DNA fragments were incubated with the methylation binding protein MeCP2 (a.k.a. MBP). The methylated DNA fragments were isolated with a spin column and then amplified with PCR using promoter specific primers for gene markers of ER stress. The Tag PCR Master Mix kit from QIAGEN was used for the PCR. The PCR products were visualized through agarose gel electrophoresis and were semi-quantified by Image J after normalized against corresponding input PCR products from the genomic DNA fragments without the MeCP2 incubation. The following primer pairs were used:

Atf6, 5′-CTTCTTTAGGAGGTAAGTGCG-3′; 5′-TGAGTAACCTGAAACGGCG-3′;

Chop, 5′-AGAGAAGCGGGTGGACTATC-3′; 5′-TAACTGACCTCAAGAGCGG-3′;

Gapdh, 5′-AAGCAAAGGTTATCACCAGG-3′; 5′-TACGCCATAGGTCAGGATG-3′;

Grp94, 5′-ACTCAGAGACATTTCCCGC-3′; 5′-GAACTCACCAATCGTGCCTC-3′;

PDI, 5′-AGCCACCCAAATCTCCATC-3′; 5′-TGCTGCTCCCAGGAATAAG-3′.

For real-time PCR analysis of promoter methylation of ERAD factors, genomic DNA was extracted with Wizard^®^ Genomic DNA Purification Kit (Promega, Madison, WI, USA) from mouse liver tissues. Then the DNA was fragmented with Episonic Multifunctional Bioprocessor (Epigentek, Farmingdale, NY, USA) into average size of 400 bp with an average power delivery of 170~190 W for 40 cycles. The size and quality of the fragment were confirmed with gel electrophoresis and Nanodrop, respectively. The methylated DNA was enriched with *MethylMiner*^TM^ Methylated DNA Enrichment Kit (Invitrogen) and the resulting DNA fragments were isolated by binding to magnetic beads conjugated with methylation binding protein MeCP2 and eluted with high concentration of NaCl followed by purification with ethanol precipitation in the presence of glycogen. The promoter methylation was quantified by qPCR with ABI qPCR system and levels of methylation were calculated after normalized with input. The following primer pairs were used:

β-actin, 5′-GTTCCGAAAGTTGCCTTTTATG-3′; 5′-CAACGAAGGAGCTGCAAAGAA-3′;

Creld2, 5′-CCGATAGAAGATTACGGTTCTG-3′; 5′-CTGATGTGGACCAATTGAGG-3′;

Derl3, 5′-GATTCTAGAGTTTTACAGAATGTCA-3′; 5′ATCTAGAAAAGAACCAATAGCAAG-3′;

Herpud1, 5′-GTTCCGAAAGTTGCCTTTTATG-3′; 5′-AAATTGTGCCCTCACAAAGC-3′;

Wfs, 5′-CACACACACTTTTTGTACTCG-3′; 5′-GCTATTACAATACTGACTAAGGTC-3′;

Yod1, 5′-CCATGATGAAGTGTCTTCCTA-3′; 5′-GCTATTACAATACTGACTAAGGTC-3′.

### MICROARRAY ANALYSIS OF TRANSCRIPTIONAL EXPRESSION OF GENES

Total hepatic RNA was isolated from fresh liver tissues using the RNeasy Mini Kit from QIAGEN following the manufacturer’s instructions and with an addition of 500 U of an RNase inhibitor (RNAguard, Amersham Pharmacia Biotech) to each starting material of 300 mg. Gene profiling and analysis was performed in the Cancer Center Microarray Core Facility of Keck School of Medicine of USC using Illumina’s Sentrix MouseRef-8 V2.0 Expression BeadChip (Illumina, San Diego, CA, USA). The quality of total RNA from liver samples was evaluated using an Experion apparatus (Bio-Rad Laboratories, Hercules, CA, USA). Total RNA (0.5 μg) from each sample was labeled and the hybridized biotinylated cRNA was detected with streptavidin-Cy3 and quantitated using Illumina’s BeadArray Reader Scanner in accordance with the manufacturer’s instructions. Microarray data were processed and analyzed with the Illumina BeadStudio software. Data of the average signal was filtered with a *p*-value (<0.05) and normalized via rank invariant normalization, after which significant changes (2- to 10-folds) were clustered for ER stress pathways and exported for heat-mapping comparisons.

### STATISTICAL ANALYSIS

Values are expressed as means ± SD unless otherwise indicated. Statistical analyses were performed using ANOVA for comparison of multiple groups or the Student’s *t*-test for pair-fed groups. *p* < 0.05 was considered significant.

## RESULTS

### EFFECTS OF LONG-TERM ALCOHOL FEEDING ON LIVER TUMOR DEVELOPMENT

**FIGURE 1:**
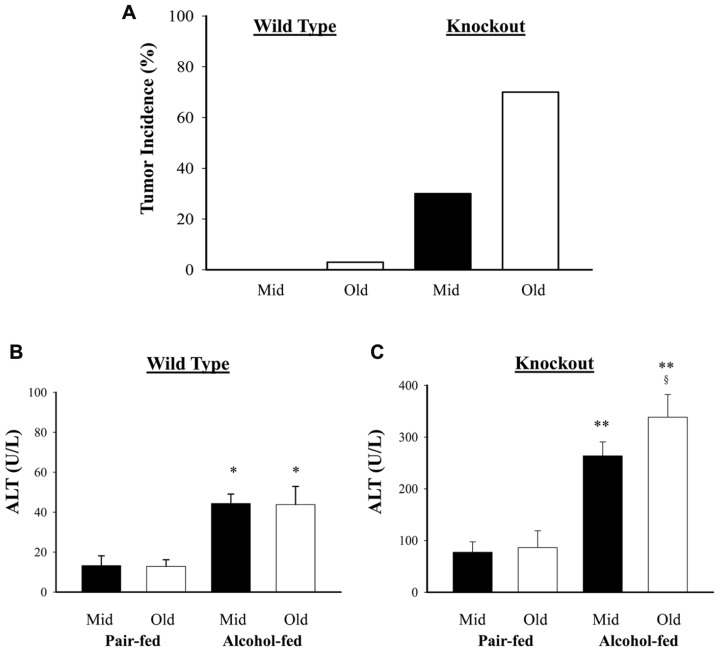
**Effects of alcohol on liver tumorigenesis in mice under constitutive endoplasmic reticulum (ER) stress.** Female mice at age of 6–8 months (Mid) and at age of 14–16 months (Old) were fed alcohol respectively for 12 months. **(A)** Liver tumor occurrence in mice fed alcohol and high fat diet; **(B)** serum alanine aminotransferase (ALT) levels in wild type mice fed alcohol; **(C)** ALT in knockout mice fed alcohol. *p < 0.05; **p < 0.01 compared to pair-fed control; ^§^*p* < 0.05 compared between mid and old age groups, *n* = 5.

**FIGURE 2:**
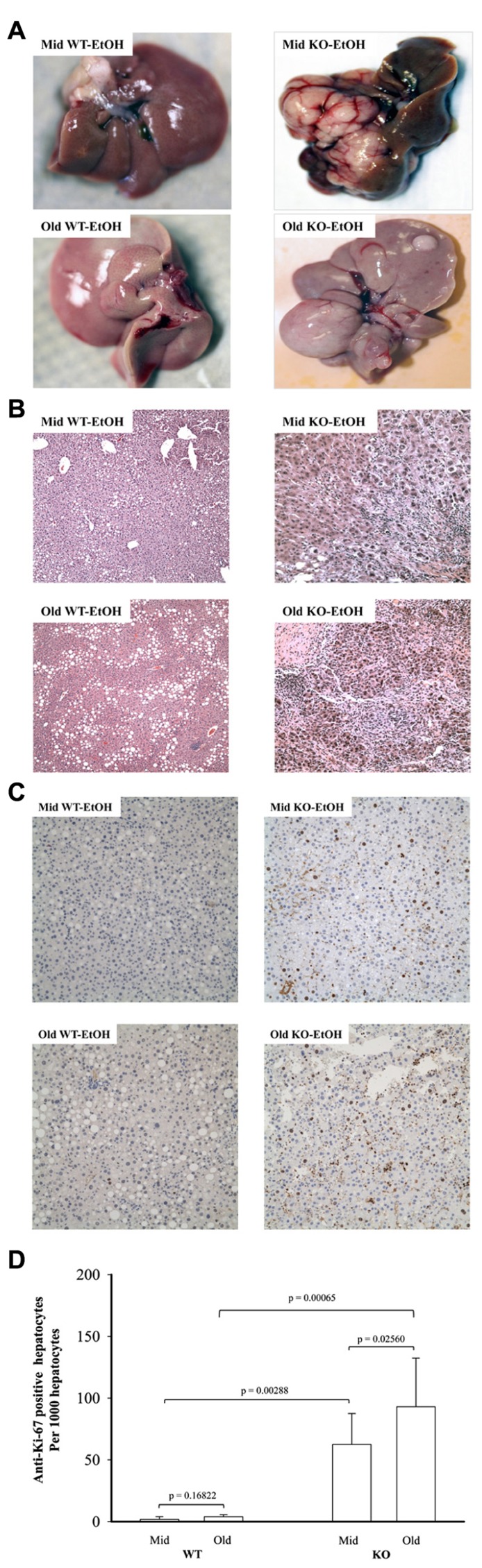
**Histology of alcohol-induced liver tumors in female mice under endoplasmic reticulum stress.** Female mice at age of 6–8 months (Mid) and at age of 12–16 months (Old) were fed alcohol (EtOH) respectively for 12 months. **(A)** Liver images showing alcohol-induced liver tumors in knockout (KO) mice. WT, wild type; **(B)** hematoxylin and eosin (H&E) staining of the liver tissues reveal alcohol-induced moderate lipid accumulation in WT mice of middle age, severe lipid accumulation in older WT mice, neutrophil infiltration and nodular formation in KO mice of middle age, and severe inflammation and multiple neoplastic hepatic lesions in older KO mice; original magnification: ×100; **(C)** liver immunohistochemistry with anti-proliferative cell nuclear antigen Ki-67 antibodies. Original magnification: ×200. **(D)** Quantitation of anti-Ki-67 positive hepatocytes.

Previous studies demonstrated that genetic ER stress predisposition with a liver-specific deletion of BiP led to fatty liver injury in both male and female mice and hepatic tumorigenesis in a significant portion of female mice at age of greater than 17 months (Ji et al., 2011; Lau et al., 2013). To know effects of alcohol on the liver tumorigenesis in the BiP KOs, we fed the mice with an alcohol HFD abbreviated as alcohol diet. Premature death was observed in greater than 50% of the KO mice fed a standard high dose of alcohol diet (6.5 g alcohol/kg body weight). Alcohol doses at less than 4 g/kg body weight were thus adopted for the experiments. At the reduced alcohol doses, liver tumors were not observed in either WT or KO males during an experimental period of 2.5 years. Thus, all subsequent studies and comparisons were focused on females. **Figure 1** demonstrates that liver tumors were observed in less than 2% of WT females fed alcohol at 12–16 months old (Old) but not in those at 6–8 months old (Mid). Alcohol induced liver tumors in 30% of the Mid female KOs and 70% of the Old female KOs (**Figure 1A**). The tumor occurrence was associated with severity of liver injury that was indicated by increased serum ALT. The alcohol feeding increased ALT by less than threefold in WT. ALT levels were constitutively higher in the KOs than in WT (**Figures 1B,C**), which were further increased by more than fivefold in response to alcohol (**Figure 1C**). Interestingly, ALT levels were significantly higher in the female KOs of older age than those of middle-aged. Histologically, mild to moderate lipid accumulation was observed in the WT females fed alcohol (**Figure 2**), which was consistent with previous findings (Ji et al., 2011). In contrast, two or more tumor masses were observed in the livers of the middle-aged female KOs fed alcohol and multiple proliferative nodules were observed in the livers of the older female KOs fed alcohol (**Figure 2**). Neutrophil infiltration was observed in the liver tumors of KOs fed alcohol. The number of Ki-67 positive hepatocytes was significantly increased in the KO compared to the WT (**Figures 2C,D**). More proliferative cells were found in the older KO mice fed alcohol than in the middle-aged KO fed alcohol (**Figure 2D**).

### EFFECTS OF CONSTITUTIVE ER STRESS ON METHYLATION OF DNA PROMOTERS OF UPR MARKERS

**FIGURE 3:**
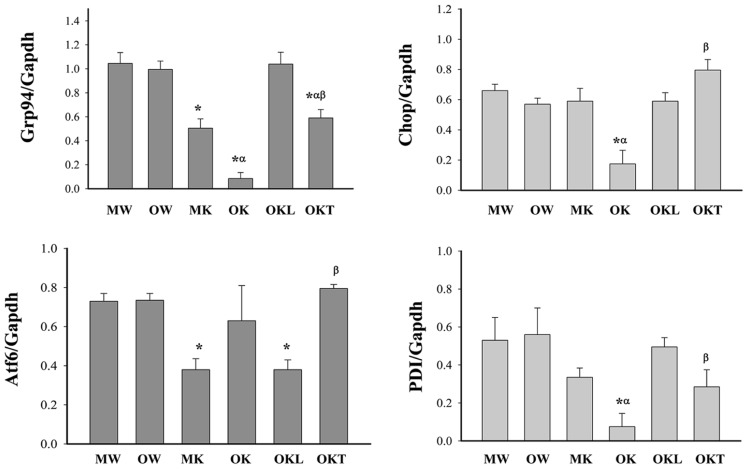
**Methylation of CpG islands of ER stress gene promoters in the livers of mice at different ages.** Grp94, glucose-regulated protein 94; Gapdh, glyceraldehyde-3-phosphate dehydrogenase; Chop, DNA damage-inducible transcript 3, also known as C/EBP homologous protein; Atf6, activating transcription factor 6; PDI, protein disulfite isomerase; MW, wild type of middle age; OW, wild type of older age; MK, BiP knockout of middle age; OK, BiP knockout of older age without liver tumors; OKL, normal liver portion from tumor bearing livers of older BiP knockouts; OKT, tumor portion from tumor bearing livers of older BiP knockouts; *p < 0.05, compared between wild type of same age; ^α^*p* < 0.05, compared between middle and older mice of same genotype; ^β^*p* < 0.05, compared between normal liver portion and tumor portion of tumor bearing livers of knockouts of same age. *n* = 3.

DNA methylation of cytosine residues at CpG dinucleotides is a commonly occurring modification of human DNA. Aberrant methylation of CpG islands is often related with cancer (Rakyan et al., 2011). Evidence is emerging for aberrant methylation of hepatic ER stress pathways (Lenz et al., 2006; Leclerc and Rozen, 2008; Esfandiari et al., 2010). In order to seek evidence to support a potential role of DNA methylation in constitutive ER stress-induced liver tumorigenesis in mice of different ages, we focused on examining the methylation of DNA promoters of selective UPR stress marker genes: Hsp90b1 (Grp94), Ddit3 (Chop or Gadd153), Atf6, and Pdia3 (PDI). The CpG island regions of Grp94, Chop, Atf6, and PDI genes were methylated in DNA isolates from the livers of WT mice of both age groups while only low and moderate levels of the DNA methylation were observed in the livers of KO mice of both age groups (**Figure 3**). There was no difference in the methylation of the DNA promoters of the UPR marker genes between the middle-aged and older WT mice (**Figure 3**). However, in KO mice without liver tumors, the methylation of Grp94, Chop, and PDI was lower in the older group than in the middle-aged group and the methylation of Atf6 was increased in the older group than in the middle-aged group. In the older KO mice with liver tumors, overall methylation of Grp94, Chop, or PDI was increased compared to middle-aged KO and there was a significant methylation difference between the normal liver portion and the tumor portion of the tumor bearing livers. The methylation of Grp94 and PDI tended to be lower in the tumor portion of KO mice with liver tumors than in the normal liver portion whereas the methylation of Chop and Atf6 tended to be higher in the tumor portion of the tumor bearing livers than the normal liver portion. These data indicate differential or abnormal methylation patterns of the UPR factors in the BiP KOs of different ages.

### EFFECTS OF LONG-TERM ALCOHOL FEEDING ON PROTEIN EXPRESSION OF ER STRESS MARKERS

**FIGURE 4:**
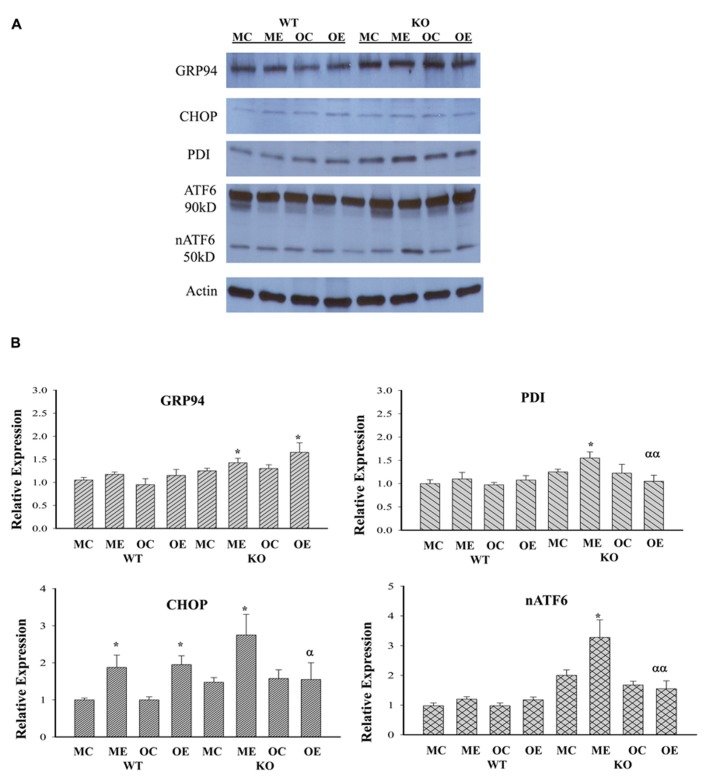
**Immunoblotting analysis of liver proteins of ER stress markers from alcohol-fed mice.** KO, liver-specific knockout of immunoglobulin heavy chain-binding protein (BiP), also known as glucose-regulated protein 78 (GRP78); WT, wild type littermate; GRP94, glucose-regulated protein 94; CHOP, DNA damage-inducible transcript 3, also known as C/EBP homologous protein; nATF6, activated form of the activating transcription factor 6; PDI, protein disulfide isomerase; MC, pair-fed wild type of middle age; ME, alcohol-fed wild type of middle age; OC, pair-fed knockout of older age; OE, alcohol-fed knockout of older age; **(A)** representative western blots of the selective ER stress markers; **(B)** relative expression of each marker protein; *p < 0.05 compared between pair-fed and alcohol-fed; ^α^*p* < 0.05, ^α^^α^*p* < 0.01 compared between alcohol-fed middle and older mice of same genotype. *n* = 3.

Although both chronic (1–2 months) and acute (1–7 days) treatments with high doses of alcohol (i.e., 6.5 g/kg body weight) have been reported to induce ER stress response that contributed to liver injury (Ji, 2012; Galligan et al., 2012; Longato et al., 2012; Ramirez et al., 2013), it is not clear whether long-term (i.e., 1 year) alcohol feeding at moderate doses induces ER stress response and contributes to the observed hepatic tumorigenesis as well. To know that, we examined protein expression of the ER stress markers: GRP94, CHOP, active ATF6 (nATF6), and PDI in the liver of WT versus KO animals of different age groups. **Figure 4** demonstrates that moderate alcohol increased GRP94 expression in the KO mice but not in the WT mice. There was no difference of GRP94 expression between different age groups treated with alcohol. CHOP that mediates ER stress-induced cell death was low abundant in the liver tissues in general and was increased in response to alcohol feeding in WT of both age groups and in KO of the middle-aged group. CHOP expression in the older KO was significantly different from that of the middle-aged KO. Both nATF6 and PDI were increased in the middle-aged KO and the inductions of nATF6 and PDI appeared to be suppressed in the older KO in response to alcohol.

### MARKED EFFECTS OF ALCOHOL ON TRANSCRIPTIONAL EXPRESSION OF GENES OF ERAD

**FIGURE 5:**
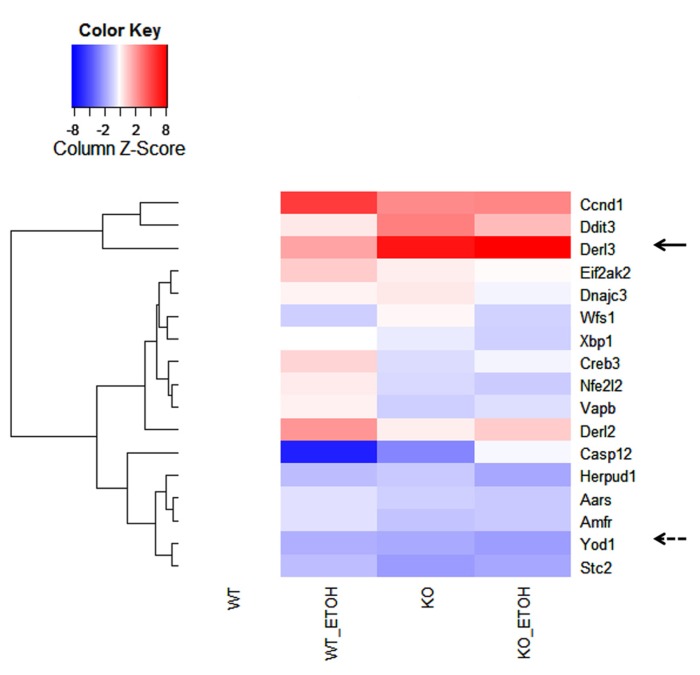
**Effects of alcohol consumption on mRNA expression of ER-associated degradation (ERAD) factors in the liver of wild type versus BiP knockout.** Ccnd1, cyclin D1; Ddit3, DNA damage-inducible transcript 3, also known as C/EBP homologous protein (CHOP); Derl2 and 3, Der1p-like protein called derlin; Eif2ak2, eukaryotic translation initiation factor 2-α kinase 2; Dnajc3, DnaJ (Hsp40) homolog, subfamily C, member 3, also known as p58IPK; Wfs1, Wolfram syndrome gene; Xbp1, X-box binding protein 1; Creb3, cAMP responsive element binding protein 3; Nfe2I2, NF-E2-related factor 2; Vapb, the vesicle-associated membrane protein B; Casp12, caspase-12; Herpud1, homocysteine-inducible, endoplasmic reticulum stress-inducible, ubiquitin-like domain member; Aars, alanyl-tRNA synthetase; Amfr, autocrine motility factor receptor, E3 ubiquitin protein ligase; Yod1, hydrolase also known as Otud2 that removes conjugated ubiquitin from proteins and participates in ERAD; Stc2, stanniocalcin 2. The solid arrow indicates strong induction by alcohol; the dashed arrow indicates strong inhibition by alcohol.

DNA microarray analysis of approximately 19,000 transcripts of known genes was further performed to identify genes that were related to UPR/ER stress and induced by the long-term moderate alcohol feeding. Three hundred eighty two transcripts were altered significantly in the alcohol-fed animals. Among them, molecular chaperones including Grp170, oxygen-regulated protein 150 (ORP150), PDI, Dnajc3 (DnaJ homolog, subfamily C, member 3, also known as p58IPK), Grp94, ERdj5 (ER-resident protein containing DnaJ and thioredoxin domains), and calreticulin; ubiquitin and protein degradation factors including Usp 4 and 18, Ube3b, EDEM2, and Der1p-like protein 3 (Derl3), transcription factors regulating apoptosis including Nupr1 (nuclear protein 1), Chop, Trib3 (tribbles homolog 3), Gadd45, and FoxO, some nuclear factor-kappaB (NFκB) targeted genes including tumor necrosis factor (TNF) related protein 1 and TNF receptor-1 (TNFR1) were increased, whereas Biklk and hepcidin 1 were decreased in response to the long-term alcohol feeding. Interestingly, the long-term alcohol feeding seemed to have strong effects on transcriptional expression of genes involved in ERAD in the KO mice (**Figure 5**). Two- to eightfold increase in derl3, Chop, and Ccnd1 (cyclin D1) was detected in the alcohol-fed KO in comparison with the pair-fed WT. Two- to eightfold decrease in Eif2ak2 (eukaryotic translation initiation factor 2α kinase 2), Wfs1 (Wolfram syndrome gene), Xbp1 (X-box binding protein 1), Creb3 (cAMP responsive element binding protein 3), Nfe2I2 (NF-E2-related factor 2), Vapb (the vesicle-associated membrane protein B), Casp12 (caspase-12), Herpud1 (homocysteine-inducible, endoplasmic reticulum stress-inducible, ubiquitin-like domain member also known as Herp), Aars (alanyl-tRNA synthetase), Amfr (autocrine motility factor receptor), E3 ubiquitin protein ligase, Stc2 (stanniocalcin 2 also known as Hrd1), and Yod1 (hydrolase that removes conjugated ubiquitin from proteins and participates in ERAD was detected in the alcohol-fed KO in comparison with the pair-fed WT.

### DEFERENTIAL EFFECTS OF LONG-TERM ALCOHOL FEEDING ON DNA METHYLATION OF ERAD FACTORS

**FIGURE 6:**
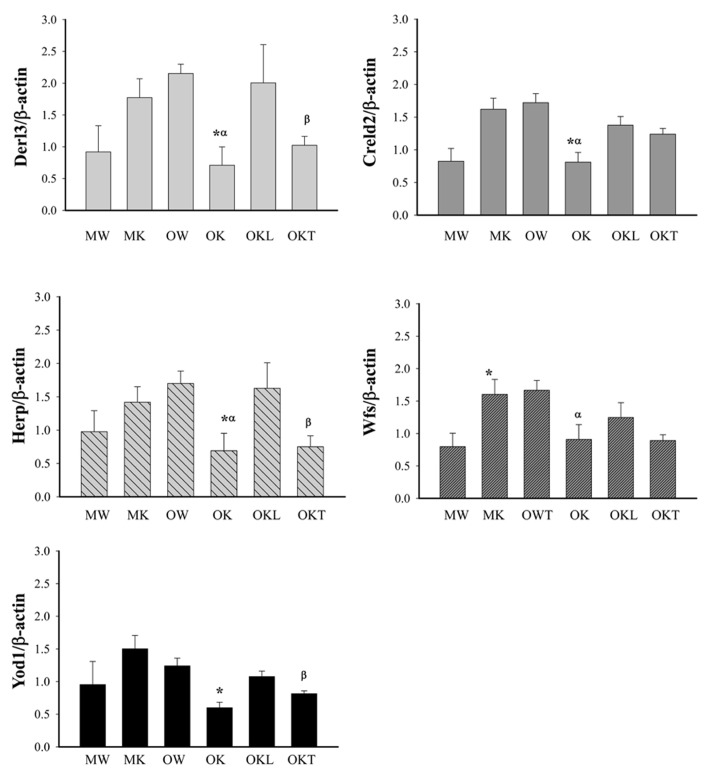
**Methylation of CpG islands of ERAD gene promoters in the liver of alcohol-fed mice of different ages.** Derl3, Der1p-like protein; Creld2, cysteine-rich with EGF-like domains 2; Herp, homocysteine-inducible, endoplasmic reticulum stress-inducible, ubiquitin-like domain member also known as Herpud1; Wfs1, Wolfram syndrome gene; Yod1, hydrolase that removes conjugated ubiquitin from proteins and participates in ERAD. MW, middle-aged wild type; MK, middle-aged knockout; OW, older wild type; OK, older knockout without liver tumor; OKL, the normal liver portion of tumor bearing livers of KO; OKT, the tumor portion of tumor bearing livers of KO. *p < 0.05, compared between wild type of same age; ^α^*p* < 0.05, compared between middle and older mice of same genotype; ^β^*p* < 0.05, compared between normal liver portion and tumor portion of tumor bearing livers of knockouts of same age. *n* = 3.

The above strong effects of long-term alcohol on the expression of ERAD prompted us to examine further methylation of the promoters of selective ERAD factors. In the middle-aged group, the methylation of the promoters of Derl3, Creld2, Herp, and Yod1 was not significantly changed in the KO compared to the WT (**Figure 6**). In contrast in the older mouse group, the methylation of the promoters of Derl3, Creld2, Herp, Wfs, and Yod1 was lower in the KO than in the WT. In addition, the methylation of Derl3, Herp and Yod1 was increased in the normal liver portion of tumor bearing livers of older KO compared to older KO without liver tumors. In the tumor bearing livers, the methylation of Derl3, Herp, and Yod1 was reduced in the tumor portion compared to the normal liver portion whereas the methylation of Creld2 or Wfs was not significantly changed in the tumor portion compared to the liver portion. The data suggest a potential association of impaired methylation of the ERAD factors in the livers of BiP KOs with aging and liver tumor development.

### CO-OCCURRENCE OF ALTERED ERAD AND TUMORIGENESIS SIGNALING IN THE LIVER OF STRESSED MICE

**FIGURE 7:**
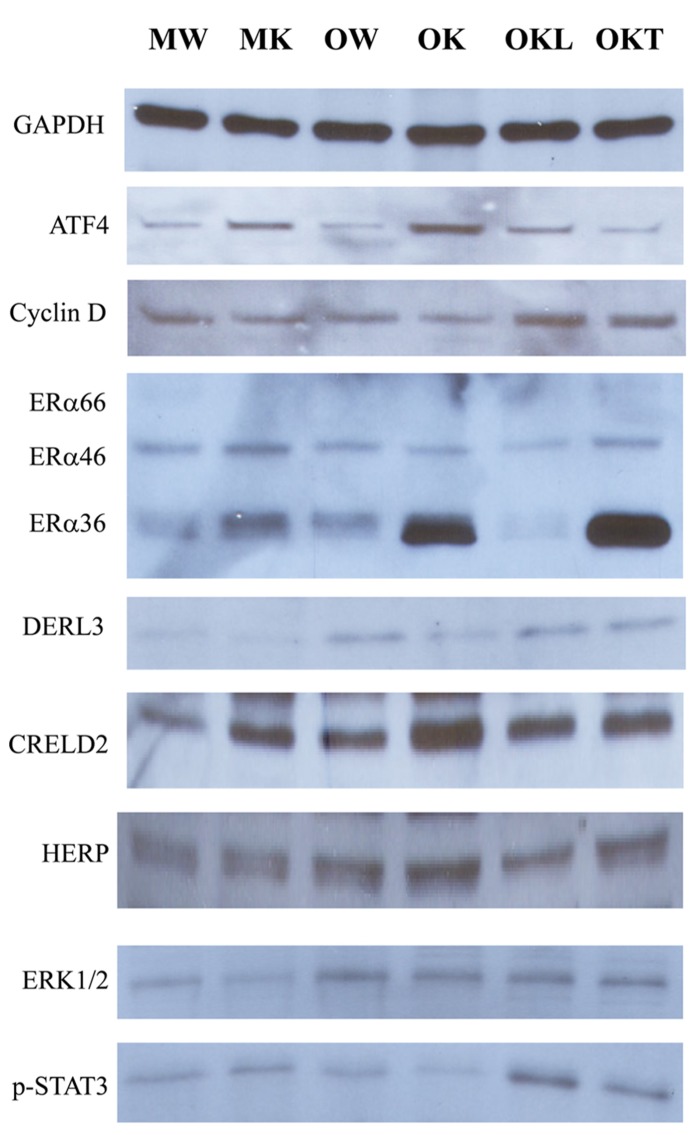
**Effects of alcohol and aging on ERAD and tumorigenesis pathways.** ERα, estrogen receptor α variants (ERα36, 46, and 66); p-ERK1/2, phosphorylated extracellular signal-regulated protein kinases 1 and 2; p-STAT3, phosphorylated signal transducers and activators of transcription; MW, middle-aged WT; MK, middle-aged KO; OW, older WT; OK, older KO; OKL, the liver portion of tumor bearing livers of older KO; OKT, the tumor portion of tumor bearing livers of older KO.

**FIGURE 8:**
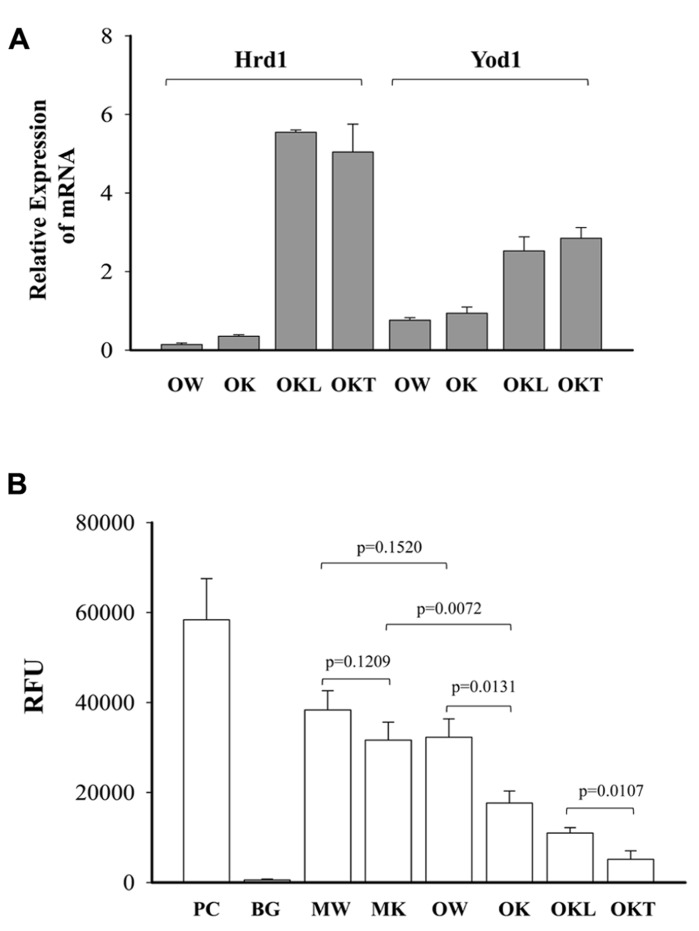
**Effects of alcohol and age on mRNA expression of ERAD factors and proteasome activities.** **(A)** Relative expression of mRNA of Hrd1 (synoviolin, an E3 ubiquitin ligase) and Yod1 analyzed with quantitative PCR and normalized with Gapdh; **(B)** comparison of 20S proteasome activities between wild type (WT) and knockout (KO). Data are presented as RFU (relative fluorescent unit) recorded with a fluorometer, *n* = 5. PC, positive control from analysis kit; BG, negative background; MW, middle-aged WT; MK, middle-aged KO; OW, older WT; OK, older KO; OKL, the liver portion of tumor bearing livers of older KO; OKT, the tumor portion of tumor bearing livers of older KO.

From our previous research with feeding of a diet contained much higher purified fat, we found both ERK (the Ras-dependent extracellular signal-regulated kinase) and Jak-Stat pathways were likely involved in stress induced liver tumorigenesis in this KO model (Lau et al., 2013). To know also whether the ERAD alterations by long-term alcohol feeding activate the two signaling pathways of liver tumorigenesis, we examined protein expression of ERAD and phosphorylation of ERK1/2 and STAT3. Increased expression of the transcription factor-ATF4 was detected in the liver of both middle-aged and older KOs (**Figure 7**). However, ATF4 was inhibited in the tumor portion compared to the liver portion. Cyclin D was slightly inhibited in all KOs compared to WT. Consistent with previous findings, ERα36 (estrogen receptor α variant 36) was increased in the middle-aged KO and was greatly increased in older KO and in the tumor portion. DERL3 was increased in both older and the tumor portion. CRELD2 was increased in both middle-aged and older KOs. The expression pattern of HERP was similar to CRELD2. Phosphorylation of ERK1/2 was detected in older WT and KOs whereas phosphorylation of STAT3 was observed only in the KOs with liver tumors (**Figure 7**). The mRNA expression of Hrd1 and Yod1 was increased by three- to sixfold in the older KO with liver tumors (**Figure 8A**). In addition, the 20S proteasome activities were reduced by 44% in older KO compared to middle-aged KO, by 45% in older KO compared to older WT, and by 53% in the tumor portion compared to the liver portion (**Figure 8B**). There were no significant differences in the proteasome activities between middle-aged KO and middle-aged WT or between middle-aged and older WT.

## DISCUSSION

Alcohol consumption is well known to be a risk factor for chronic liver disease, from steatosis or fatty liver to steatohepatitis to fibrosis to cirrhosis and even liver cancer (HCC; Gao and Bataller, 2011; Brandon-Warner et al., 2012; Testino et al., 2012). Alcohol attributes to cancer related death significantly (Morgan et al., 2004). Alcohol metabolism directly contributes to the initiation of cancer. For instance, the first metabolite of alcohol-acetaldehyde is highly reactive, forming DNA-acetaldehyde adducts that can incorporate into the genome, leading to mutagenesis and transformation of healthy cells into tumor cells. Alcohol consumption induces CYP2E1 and results in the production of ROS, directly damaging DNA or generating lipid peroxidation products capable of forming mutagenic DNA adducts. ROS promotes inflammatory environments damaging to healthy host tissue leading to the development of cancer through mutagenesis (Jerrells, 2012). Alcohol-induced organelle stress, especially ER stress has been associated with a spectrum of liver diseases (Dara et al., 2011). Evidence for ER stress-induced hepatic tumorigenesis is emerging (Wang et al., 2010; Lau et al., 2013). However, how alcohol influences ER stress and liver tumorigenesis is not clear. Our current study using the animal model with a liver KO of the chaperone BiP and under constitutive hepatic ER stress may reveal a few critical clues with respect to alcohol-induced cancer. First, the long-term moderate alcohol-induced liver tumor development was observed only in the ER stress-predisposed KO animals. This suggests that additional insults such as genetic and environmental stresses may be required for the alcohol-induced hepatic tumorigenesis. This can also explain why, to date, no rodent model has demonstrated the formation of HCC in the setting of chronic alcohol consumption alone. Alcohol-induced HCC is often reported under circumstantial conditions such as an alcohol-preferring (P) rat line that voluntarily drinks large quantities of alcohol (Yip-Schneider et al., 2011), in combination with obesity (Thompson et al., 2013) or co-dosing with carcinogenic diethylnitrosamine (DEN; Brandon-Warner et al., 2012), in the presence of expression of hepatitis C virus (HCV) components (Machida et al.; 2009), or consuming alcohol for an excessively long period of more than 70 weeks (Tsuchishima et al., 2013). While some of the observations reported by others may not be clinically relevant, our results support the concept of necessity of additional insults for the alcoholic HCC development and are significant since alcohol-induced ER stress occurs in human alcoholics and emerging evidence has already demonstrated that polymorphic responses (SNPs) of BiP are associated with alcohol, HCC, and other types of cancer in the human population (Zhu et al., 2013).

Second, alcohol-induced ER stress and liver cancer may also depend on aging of this animal model. We found in the present study that a tendency for liver cancer development was higher in ER stress-predisposed (KO) mice fed alcohol at older age (12–16 months) than at middle age (4–6 months). Remarkably robust and consistent impacts on ALT levels and the ER stress were detected in the older mice. Long-term alcohol apparently suppressed the protective UPR, i.e., inhibition of GRP94, PDI, and ATF6 and promoted ER stress-mediated elimination of injured cells, i.e., increase of CHOP. Aging might deteriorate the shift from adaption by the UPR to injury by alcohol. The underlying mechanism is currently not known and may be complex. In other systems, aging had a prominent role in determining genomic DNA methylation and aberrant methylation of CpG islands has often been related with cancer (Rakyan et al., 2011; Ozen et al., 2013). Alcohol is known to affect DNA methylation by its interference with one carbon metabolism and by alteration of the methylation of specific promoters (Medici and Halsted, 2013; Ozen et al., 2013). In relevant to the animal model with constitutive ER stress, we assumed that aging might impair methylation of DNA promoters of the UPR components. As we expected, there was no difference in the methylation of the DNA promoters of the UPR marker genes between the middle-aged and older WT mice (**Figure 3**). However, differential effects of alcohol on the methylation of ER components were observed in the KO mice. The methylation of Grp94, Chop, and PDI was lower in the older KO group than in the middle-aged KO group whereas the methylation of Atf6 was higher in the older KO group than in the middle-aged group. In addition, there were significant differences between older KO with and without liver tumors and between normal liver portion and tumor portion of tumor bearing livers. For instance, hypomethylation of Grp94 and hypermethylation of Chop were seen in the tumor portion of older KO mice, which were respectively consistent with increased protein expression of GRP94 and decreased protein expression of CHOP in the tumors. Thus, our findings indicate that alterations of methylation patterns of the UPR/ER stress factors in the aging BiP KOs are likely contribute to liver tumor development.

Third, proteins that fail to fold and assemble into their mature forms are usually removed by the ERAD process that depends on activities of ubiquitin and proteasome. Although it is not clear based on the current data whether the methylation of UPR causally influences the methylation of ERAD or vice versa, the constitutive ER stress in the liver of animals without the chaperone BiP must burden the ERAD, which may be worsened by additional stress such as altered cellular levels of *S*-adenosyl-L-methionine (the principal biological methyl donor) as a consequence of chronic alcohol consumption (Kharbanda, 2013). We support this assumption with the observations that the effects of the long-term alcohol on transcriptional and translational expression of the ERAD related genes including derlin 3, Creld2, Herpud1, and Wfs1 were stronger than on the expression of the UPR related genes such as Chop, cyclin D, and Xbp-1. The alterations of ERAD expression corresponded to decreased proteasome activities and were age-related. In the middle-aged groups, methylation of the promoters of Derl3, Creld2, Herpud1, and Yod1 was not altered significantly in the KO than in the WT (**Figure 6**) whereas in the older mouse groups, the methylation of the promoters of these ERAD genes was lower in the KO than in the WT. Particularly, the methylation of Derl3 and Herp was reduced in the tumor portion of older KOs, methylation of Creld2, Wfs, and Yod1 was not changed in the normal liver portion, and mRNA expression of Hrd1 and Yod1 was remarkably increased in both liver and tumor portions of KO with liver tumors. These differential effects of alcohol and aging on the ERAD factors may reflect a severe impairment of protein processing in the liver under long-term stress. Therefore, we speculate that long-term alcohol has profound effects on protein quality control in aging animals, which in general, affects protein turnover leading to accumulation of excessive unfolded proteins, which continuously stimulates pathological changes leading to tumorigenesis. One identified potential tumorigenic factor in this study is the abundant estrogen receptor α variant ERα36, which might result either from malfunctioning of proteasomal degradation, impaired physical interactions between cyclin D and the authentic ERα, or alternative splicing of internal exons of ERα (Zwijsen et al., 1997; Fu et al., 2004; Rao et al., 2011; Lau et al., 2013). ERα36, perhaps together with other improperly processed proteins yet to be identified, interfered with phosphorylation of ERK1/2 and STAT3 in the older KO female fed alcohol resulting in high incidence of tumors. The exact molecular mechanisms up and downstream of ERα36 pertinent to UPR/ER stress signaling or abnormal methylation await further investigations.

Fourth, there are reports that human males are more likely developing HCC than females in some regions of the world (Venook et al., 2010; Center and Jemal, 2011). However, the male prevalence of HCC is circumstantial and not contradictory to our findings for a couple of reasons. The male prevalence usually occurs in areas such as Asia where men tend to expose themselves more to additional HCC risk factors such as hepatitis B virus (HBV) and aflatoxin from contaminated maize and peanut. The other reason is that higher levels of estrogen in young- and middle-aged females may play some protective role against HCC development, which might be age-dependent. There are epidemiological data demonstrate that the incidence of HCC drops significantly in old individuals of both genders (El-Serag, 2011). Since the age range of the experimental animals of this study corresponds to humans aged of greater than 50, which is generally a post-menopause age for women, the possible protective effects of estrogen are diminishing and there should be equal odds of HCC development for aged men and women without additional gender-specific risks. In this respect, the impaired expression of estrogen receptor α caused by long-term ER stress in females consists of a gender-specific risk and is most likely responsible for the high incidence of liver tumors observed in aged females.

In summary, in ER stress-predisposed older animals fed alcohol for a prolonged period, we observed marked alterations in expression and promoter methylation of ERAD genes that were co-present with development of liver tumors. We propose that long-term alcohol consumption and aging may promote liver tumorigenesis through interfering with DNA methylation and expression of genes related to the ERAD.

## Conflict of Interest Statement

The authors declare that the research was conducted in the absence of any commercial or financial relationships that could be construed as a potential conflict of interest.
